# Ocular Manifestations of Inflammatory Bowel Disease

**DOI:** 10.7759/cureus.40299

**Published:** 2023-06-12

**Authors:** Erik Licona Vera, Catalina Betancur Vasquez, Juan Sebastian Peinado Acevedo, Tatiana Rivera Bustamante, Jose Miguel Martinez Redondo

**Affiliations:** 1 Gastroenterology, Universidad CES, Medellin, COL; 2 Ophthalmology, Universidad del Norte, Barranquilla, COL; 3 Dermatology, Universidad de Antioquia, Medellin, COL; 4 Ophthalmology, Universidad del Sinú Seccional Cartagena, Cartagena, COL

**Keywords:** scleritis and uveitis, episcleritis, ocular, ulcerative colitis, crohn’s disease, inflammatory bowel disease

## Abstract

Inflammatory bowel disease (IBD) is a chronic disease connected to the immune system, with a predilection for the gastrointestinal tract. However, a large proportion of the patients have extraintestinal manifestations (EIM), and the ocular system is affected in some patients. The clinical presentation of ocular pathology is broad, ranging from asymptomatic cases to blindness, leading to high morbidity. Ocular complications can be primary and, in general, are associated with episodes of acute flare-ups that subside with immunological management of the digestive disease. Secondary complications arise from the primary ones and as adverse effects of immunological treatment. In addition, on many occasions, the ocular manifestation may appear prior to the presentation of digestive symptoms. The presence of a multidisciplinary team that includes a gastroenterologist and an ophthalmologist is important in order to achieve early diagnosis of ocular complications, thereby preventing, treating, and avoiding unfavorable and irreversible long-term ocular sequelae.

## Introduction and background

Inflammatory bowel disease (IBD) is a chronic, inflammatory gastrointestinal disorder of unknown etiology. It includes both ulcerative colitis (UC) and Crohn's disease (CD) [1]. These are the two most frequent phenotypes of the disease, both having a chronic course with acute exacerbations. However, their pathophysiology and clinical presentations are different [1,2].

The epidemiology of IBD is highly variable depending on the population studied. The global prevalence is estimated at 146.9 cases per 100,000 inhabitants, with an increasing incidence, especially in recently developed countries of South America, Asia, and the Middle East. However, Europe has the highest number of cases worldwide. The estimated prevalence for UC and for CD is 505 cases and 322 cases per 100,000 inhabitants, respectively [2,3].

The origin of IBD is complex and multifactorial, including genetic, infectious, environmental, and immunological factors [4,5]. Some authors describe a genetic origin of the disease, based on its higher prevalence in European countries and North America compared to other regions. It has been related to variations in the *NOD2* gene (Nucleotide-binding oligomerization domain containing 2), which is located on chromosome 16. This gene can modify the innate immune response to intestinal flora, increasing the risk of developing IBD [6]. However, the main factor for the development of IBD is immune system dysregulation, the increased production of tumor necrosis factor-alpha (TNF-α), and interferon-gamma. The immunological etiology of IBD is supported by its positive response to immunosuppressive therapy [1].

The European Crohn's and Colitis Organization (ECCO) proposed an operational definition of extraintestinal manifestation (EIM) pathology in a patient with IBD and for which the pathogenesis may depend on the extension/translocation of immune responses from the intestine or, it may be an independent inflammatory event, but perpetuated by IBD or sharing genetic or environmental predisposition with IBD; in order to provide a frame of reference for scientific discourse [7].

The ocular involvement in UC is 12-35% compared to CD, which is 25-70% [6,8]. Between 6-40% of patients with IBD have two or more EIMs, which has a negative impact on quality of life and significantly increases morbidity [1,2]. EIMs occur in 25% of patients before the diagnosis of IBD, while in the remaining 75% of patients, they occur during or after being diagnosed with the disease (3). The most frequent extraintestinal manifestations are osteoarticular, mucocutaneous, and ophthalmological; however, any non-digestive organ may be compromised [8]. The differences in ocular manifestations in UC and CD are given in Table 1.

**Table 1:** Differences in Ocular Manifestations between Ulcerative Colitis and Crohn's Disease.

Ocular Manifestations	Ulcerative Colitis	Crohn`s Disease
Anterior Uveitis	Yes	Yes
Episcleritis	Yes	Yes
Scleritis	No	Yes
Conjunctival disorders	No	Yes
Dry eye syndrome	No	Yes

EIMs predict and are associated with gastrointestinal involvement in IBD. For example, peripheral arthritis, erythema nodosum, aphthous stomatitis, Sweet's syndrome, and episcleritis are associated with gastrointestinal activity in IBD. However, uveitis and ankylosing spondylitis can occur in the absence of gastrointestinal activity. Additionally, additional manifestations such as sclerosing cholangitis and pyoderma gangrenosum may also be present. Gastrointestinal activity can vary [3,9].

The main risk factors for the development of EIMs are being under 40 years old, being female, the use of biological therapy, having a family history of IBD, and having extensive digestive involvement. Additionally, the presence of any EIM predisposes to the appearance of others in different distant organs [8].

Ocular manifestations are the third most frequent IEM, after joint and mucocutaneous involvement. The prevalence of ocular involvement varies according to the population group studied and the presence or absence of symptoms [8,10]. They can be secondary to IBD per se, immunosuppressive drugs, or other factors such as age and the comorbidities of each patient [11-13]. The objective is to conduct a narrative review of the available literature on ocular involvement secondary to IBD, as some of these manifestations result in significant visual morbidity, which can even lead to blindness. Hence, it is important to understand the relationship between IBD and the ocular system, its risk factors, the clinical presentation, and the behavior of the most frequent ocular manifestations.

A literature search was conducted in PubMed from January 10th to August 8th, 2022, using the following search terms: inflammatory bowel disease, Crohn's disease, ulcerative colitis, ocular, episcleritis, scleritis, and uveitis. Additionally, the reference management software ZOTERO 5.0 (2006; Corporation for Digital Scholarship, Vienna, Virginia, United States) was used to identify any additional articles not identified in the aforementioned literature searches. Studies published until August 2022 were included and added with the assistance of the reference manager. Data extraction was done manually. The bias of the studies was assessed based on the information provided in each publication, and articles related to pregnant women and children were excluded. Subsequently, we focused on relevant studies for the stated objective.

## Review

The approach to ophthalmological involvement in IBD must begin by promptly identifying the clinical manifestations that lead to severe pathologies that require timely management to avoid sequelae (Table 2) [2]. In general, routine evaluation of ophthalmological involvement is recommended in all patients, even in the absence of symptoms, since it is possible to observe subclinical involvement and it is a priority before considering other treatments. Additionally, all patients with chronic glucocorticoid use should be monitored for the risk of cataracts and glaucoma.

**Table 2:** Indications for Evaluation in IBD by Ophthalmologists. Information from: Troncoso et al., 2017 [2] IBD: inflammatory bowel disease

Indications for urgent evaluation by ophthalmology.
Decreased visual acuity
Foreign body sensation
Pupillary disorder
Headache associated with visual symptoms
Corneal opacity
Red eye

EIMs of IBD are common, reaching a prevalence of up to 35% in UC and up to 70% in CD. The visual system is one of the most commonly affected extraintestinal locations, and these are simultaneously associated with skin and joint EIMs [11,14]. Algaba et al. reported in the Spanish ENEIDA registry (Nationwide study on genetic and environmental determinants of inflammatory bowel disease) with more than 31,000 patients a prevalence of ocular involvement of 2.1%, but it can range between 0.3% and 13%, being higher in patients with CD (3.5-6.8%) than with UC (1.6-5.4%) [15]. The majority of patients have active gastrointestinal involvement at the time of documenting ophthalmological involvement [2,11,16].

The spectrum of ophthalmological involvement is very broad, including the primary EIMs of IBD and secondary ophthalmopathy, related to the use of disease controller medications. According to Guillo et al, in their systematic review, episcleritis and uveitis are the most frequent ocular manifestations with 88.5%, being more prevalent in the population with CD (68.2%) than with UC (31.8%) [17]. Among the unusual manifestations are retinal vasculitis, papillitis, corneal infiltrates, perforating scleromalacia, optic neuritis, and myositis [17,18]. The pathophysiology of ocular involvement is not yet well established; however, the most accepted theory is based on the production of antigen-antibody immune complexes in the intestinal wall, which then travel through the bloodstream and affect different systems [2]. 

Primary ocular manifestations

*Episcleritis* Episcleritis is the inflammation of the episclera and it is the most frequent ocular manifestation of IBD. It is an entity that is directly related to the activity of the disease and its acute outbreaks. It should be suspected in patients with IBD who present with acute redness of one or both eyes, associated with mild ocular discomfort, irritation, or burning sensation. On ophthalmologic examination, it presents with reddish scleral patches with areas of white sclera between the dilated episcleral vessels. The topical use of phenylephrine causes blanching of the episcleral vessels in episcleritis [7]. The control of systemic disease is the most important aspect of treatment; however, topical steroids and local cold compresses are also used. In refractory cases, infliximab can be used. Caution should be exercised with the use of non-steroidal anti-inflammatory drugs (NSAIDs) because they can worsen the underlying disease, and if required, the use of cyclooxygenase 2 (COX2) is preferred [15,16].

*Uveitis* Uveitis corresponds to the acute inflammation of the uveal tract or middle layer of the eye, which includes the iris, ciliary body, and choroid. Uveitis is classified as anterior, middle, and posterior depending on the anatomical site involvement. Anterior when it affects the anterior chamber (iritis, iridocyclitis, anterior uveitis), intermediate when the vitreous is involved (pars planitis, posterior uveitis, vitritis), and posterior when it affects the retina and choroid (choroiditis, retinitis, choriorretinitis, neuroretinitis) [16]. In patients with IBD, uveitis involvement is usually anterior and is not associated with gastrointestinal tract activity. In a cohort study published in 2011, it was found that activity in CD is related to the presence of uveitis, a finding that cannot be extrapolated to UC [11]. Similar behavior has been observed in other publications [16,17]. In the study by Rogler et al., it was observed that in patients with CD, uveitis occurs in 12.2% when there is activity and in 5.2% when there is no activity [16]. On the contrary, for UC, 4.1% vs 3.5% in patients with and without activity, respectively. The typical clinical manifestations in anterior uveitis are photophobia, pain, redness, variable decrease in visual acuity, and tearing. The ophthalmological examination reveals ciliary hyperemia, hyperemia around the cornea (perikeratic), miosis, and exudation in the anterior chamber (Tyndall phenomenon). Occasionally, precipitation on the cornea and synechiae can be observed [19,20].

The treatment of this entity is based on the use of topical and cycloplegic corticosteroids. If there is no response, therapy can be escalated to systemic steroids, immunosuppressants, or anti-TNF agents [15]. In general, the prognosis is favorable; however, there may be complications such as the formation of intraocular adhesions due to chronic inflammation, which can lead to glaucoma and intraocular synechiae [7]. *Scleritis*

Scleritis is a serious ocular condition characterized by inflammation of the deep blood vessels in the sclera. It occurs in less than 1% of cases and can lead to permanent vision loss, so it is important to identify and treat it promptly. Symptoms include ocular redness, sensitivity to touch, and intense pain [5].

Clinically, it manifests as reddish or purplish patches on a continuous scleral redness background. In addition, the scleral vessels do not blanch with the application of topical epinephrine. This condition is potentially dangerous and can lead to retinal detachments and inflammation of the optic nerve. Therefore, it must be managed aggressively with systemic steroids, NSAIDs, and immunomodulators to prevent visual loss, and the patient must always be referred to an ophthalmologist [6,7]. *Corneal Disease and Other Rare Manifestations* The presence of corneal infiltrates is a rare EIM of IBD. It presents with ocular irritation, foreign body sensation, and pain; occasionally, there is a decrease in visual acuity. These lesions can be stained with fluorescein. Treatment is based on the use of immunosuppressants or oral steroids, avoiding their topical use due to the risk of corneal thinning [6,11,16]. There are other forms of ocular pathology associated with IBD and include marginal corneal disease, perforating scleromalacia, orbital inflammatory disease, occlusions of the central retinal artery and its branches, central retinal vein occlusion, retinal vasculitis, and optic neuritis [19,21,22]. These forms of clinical presentation, especially optic neuritis, vascular occlusions, and retinal vasculitis, can lead to irreversible eye damage if not recognized and treated promptly [6].

*Secondary Ocular Manifestations* In general, the ocular manifestations that occur simultaneously with the acute outbreaks of IBD respond to the management of the digestive disease. However, in certain instances, treatments such as steroids, immunosuppressive therapy, and anticholinergics may have side effects on the ocular system [2,7]. Systemic steroids are associated with multiple side effects, and in the ocular system, they can lead to posterior subcapsular cataracts, especially after long-term use [12]. Symptoms are usually minimal or may be asymptomatic. In addition, they are related to increased intraocular pressure (IOP), increasing the risk of open-angle glaucoma; however, this is more frequently associated with the use of topical steroids [7]. Immunosuppressive therapy can cause optic neuritis, ophthalmoplegia, and nystagmus, especially with the use of cyclosporine and methotrexate. The latter can also lead to irritation of the eyelids, cornea, and conjunctiva [2,11].

The relationship between ocular manifestations and IBD activity is given in Table 3. In general, the visual prognosis in patients with ocular pathology associated with IBD is favorable, as long as timely diagnosis and treatment are carried out [19].

**Table 3:** Relationship Between Ocular Manifestations and IBD Activity.

Ocular manifestation	IBD activity
Episcleritis	Associated
Scleritis	Associated
Uveitis	Associated
Conjunctival disorders	Not associated
Corneal disorders	Not associated
Dry eye syndrome	Not associated

## Conclusions

IBD should be recognized as a condition that affects multiple organs and is not limited exclusively to the digestive system. Ocular manifestations are frequent and can be the first manifestation of IBD, emphasizing the importance of conducting a comprehensive evaluation of these patients.

In patients with a previous diagnosis of IBD, the presence of ocular symptoms such as ocular redness or other signs should be considered a signal to assess the presence of ophthalmopathy and disease activity, and treatment should be adjusted appropriately. A multidisciplinary approach involving general physicians, internists, gastroenterologists, and ophthalmologists to conduct routine ocular evaluations is crucial to prevent irreversible damage.
